# *Mycoplasma pneumoniae *pneumonia revisited within the German Competence Network for Community-acquired pneumonia (CAPNETZ)

**DOI:** 10.1186/1471-2334-9-62

**Published:** 2009-05-13

**Authors:** Heike von Baum, Tobias Welte, Reinhard Marre, Norbert Suttorp, Christian Lück, Santiago Ewig

**Affiliations:** 1 Institute. for Medical Microbiology and Hygiene, Ulm University Hospital, Ulm, Germany; 2 Department of Pneumonology, Hannover University Hospital, Hannover, Germany; 3 Hospital Administration, Ulm University Hospital, Ulm, Germany; 4 Department of Infectious Diseases and Pulmonary Medicine, Charité, Berlin, Germany; 5 Institute of Med. Microbiology and Hygiene, Dresden University Hospital, Dresden, Germany; 6 Department of Pneumonology, Thoraxzentrum Ruhrgebiet, Herne und Bochum, Germany

## Abstract

**Background:**

Currently, broad empiric antimicrobial treatment including atypical coverage is recommended for patients with mild to moderate community-acquired pneumonia (CAP). Therefore, the relative impact of each atypical pathogen, particularly *Mycoplasma pneumoniae *deserves renewed attention.

**Methods:**

Based on prospective data from 4532 patients with CAP included in the German CAP-Competence Network (CAPNETZ), we studied the incidence, clinical characteristics, and outcome of patients with *Mycoplasma pneumoniae *pneumonia (MPP). The diagnosis of MPP was based on a positive PCR from respiratory samples and/or a positive IgM-titer from an acute phase serum sample.

**Results:**

307 patients (6.8%) had definite MPP (148 with positive PCR, 204 with positive IgM, 46 with positive PCR and IgM). Compared to patients with other definite and unknown etiologies, patients with MPP were significantly younger (41 ± 16 versus 62 ± 17 and 61 ± 18 years), had fewer co-morbidities, presented with a less severe disease, showed a lower inflammatory response in terms of leukocyte counts (median 8850 versus 13200 and 11000 μL) and CRP values (60 versus 173 and 73 mg/L), and had better outcomes, including a shorter length of hospitalization (9 ± 5 versus 14 ± 11 and 12 ± 9 days), fewer patients requiring mechanical ventilation (0.3 versus 4.5 and 2.1%), and a minimal mortality (0.7 versus 8.7 and 6.5%).

**Conclusion:**

In this large series of patients with definite MPP according to very strict criteria, MPP appears as a condition with a high incidence, quite specific clinical presentation, and a largely benign course. In view of a widely favorable clinical outcome, recent recommendations including regular coverage of atypical pathogens in patients with mild to moderate CAP might be reconsidered for patients in Germany as well as in other countries with comparable epidemiological settings.

## Background

*Mycoplasma pneumoniae *as an important pathogen of community-acquired pneumonia (CAP) is only rarely diagnosed in routine practice. This is explained by the many limitations of paired serology which still is the applied diagnostic tool in most cases. In several epidemiologic studies of CAP, largely relying on paired serology, *Mycoplasma pneumoniae *was identified in 5–15% of cases, resulting in a second or third rank pathogen causing CAP in most series [1,2]. Together with *Legionella spp*. and *Chlamydophila pneumoniae*, these three pathogens are usually addressed as "atypical bacterial pathogens", and considered as prominent targets for broad spectrum antimicrobial treatment including atypical coverage [3].

This approach is questionable for several reasons. First, it may discourage clinical services to include microbial investigations in their diagnostic work-up and thereby promote the decline of any effort to design targeted antimicrobial treatment. Second, relying on paired serology for the diagnosis of MPP necessarily misses acute deaths from pneumonia, and may thereby provide misleading clinical descriptions of the disease and underestimate the prognostic implications of this pathogen. Finally, lumping together all three atypical pathogens throughout all pneumonia severities at admission may heavily bias the potential prognostic implications of the pathogens included in this group, and thereby lead to recommendations of initial empiric antimicrobial treatment implying frank overtreatment.

For these reasons, based on the large CAPNETZ database, we aimed at identifying patients with CAP due to *Mycoplasma pneumoniae *using a very strict methodology, in order to revisit the epidemiology, clinical characteristics, and the outcome of these patients. In addition, we thought to reconsider the recommended diagnostic approach to atypical pathogens on the background of our findings.

## Methods

### Patient Population

A detailed description of the CAPNETZ methodology is given elsewhere [4]. In short, the inclusion criteria for the CAPNETZ study were age ≥ 18 years, the presence of a new infiltrate in chest radiography, and at least one of the following criteria: history of fever (≥ 38.3°C), cough, presence of purulent sputum or focal chest signs on auscultation. Patients who had been hospitalized during 28 days preceding the study, with severe immunosuppression or active tuberculosis were excluded.

The study was approved by the ethical review board, and all patients included gave informed consent.

### Data Collection

In this prospective study, all demographic, clinical and diagnostic data of the patients were recorded using standardized web-based data sheets created by 2 mt^® ^Ulm, Germany. The study period comprised 55 months starting on 1^st ^June 2002 and ending 31^st ^December 2006, thus including almost five autumn-winter seasons.

### Microbiological Processing and Examination

Physicians were asked to provide a sputum sample from all study patients. However, if the patient was not able to produce a sputum sample, it was the physician's decision to perform more invasive procedures. Methods applied were described previously [5]. In short, sputum and/or other respiratory secretions were immediately processed in the participating local microbiological laboratories according to the German Quality Standards in Clinical Microbiology and Infectious Diseases MIQ [6]. The results of virus testing are not included in this report.

### Investigation for *Mycoplasma pneumoniae *and case definition for Mycoplasma pneumonia

Respiratory specimens and acute-phase serum were collected, stored frozen for a maximum time period of 3 months and then sent to the central service unit in Ulm on dry ice. After arrival at the central service unit completeness of the number and kind of specimens was checked and specimens were stored at -80°C. Both respiratory specimen as well as sera were analysed retrospectively. Physicians were not aware of the test results in time to change therapy. The DNA from the clinical samples was extracted by using the QIAamp DNA Mini Kit (Qiagen, Hilden, Germany) according to the manufacturer's instructions [7].

Detection of Mycoplasma DNA from respiratory samples was performed in the Institute for Medical Microbiology and Hygiene, Dresden, the German Consulting Laboratory for *Mycoplasma pneumoniae *by using a real-time PCR targeting the inter-repetitive region of the P1 gene. We used an antigen enriched with P1 (160 KD) mature adhesin. The real-time PCR readily detects all subtypes and variants of *Mycoplasma pneumoniae *with a detection limit of approximately 10 genomic equivalents [8]. To exclude an inhibition each sample was spiked with 1000 DNA copies and retested under the same conditions.

For the detection of *Mycoplasma pneumoniae*-specific IgG, IgA or IgM antibodies the Virotech EIA (Genzyme Virotech, Russelsheim, Germany) was used according to the manufacturer's instructions. The EIA is based on a defined antigen mix that includes the P1, P100, and P30 proteins.

In the present study, definite *Mycoplasma *pneumonia was defined as: 1) a positive PCR based detection of *Mycoplasma pneumoniae *DNA in respiratory samples or 2) a positive IgM test in the acute phase serum sample.

### Statistical analysis

Comparisons between groups were performed by means of the Chi square test for categorical variables or analysis of variances (ANOVA) for continuous variables including multiple comparisons. All analyses were performed with SPSS software (SPSS 10.0, Chicago, IL). All tests of significance were 2-tailed, and alpha was set at 0.05. To correct for multiple testing the Bonferroni correction was applied to the variables presented in table 1 and the significance level was consecutively set as p < .001.

**Table 1:** Clinical characteristics and antimicrobial treatment of patients with *Mycoplasma pneumoniae *pneumonia (MPP), CAP due to other bacterial pathogens, and CAP with unknown etiology

Variable	CAP due to other definite bacterial pathogens n = 621	p^1^	MPP n = 307	p^2^	CAP with no known bacterial etiology n = 3604
Age (∅ ± SD)	61 ± 17 years	.000	41 ± 16 years	.000	61 ± 18 years

Male gender	58%	.000	41%	.000	56%

Outpatient/hospitalized	22%/78%	.000	56%/44%	.000	36%/64%

CRB 65 Score 0-1-2-3-4 (%)	33-46-16-4-1	.000	72-25-3-0.4-0	.000	39-43-15-2-0.4

Packyears(∅ ± SD)	32 ± 25	.000	15 ± 14	.000	29 ± 23

Pleural effusion	17%	.000	6%	.001	13%

Dyspnoea	80%	.000	69%	ns	72%

Pleural pain	47%	.000	34%	ns	42%

Confusion	11%	.000	2%	.001	8%

Oxygen requirement	57%	.000	25%	.000	41%

Malignancy	9%	.000	2%	.000	10%

Chronic respiratory disease	38%	.000	20%	.000	36%

Renal insufficiency	8%	.001	2%	.000	8%

Congestive heart failure	19%	.000	4%	.000	19%

Diabetes	20%	.000	3%	.000	16%

Respiratory sample obtained*	**42%**	.000	**58%**	.000	**30%**

Leucocytes (median)/μl	13 200	.000	8850	.000	11 000

CRP (median) mg/l	173	.000	60	.001	73

Duration of antibiotic therapy (∅ ± SD)	13 ± 6 days	.000	11 ± 4 days	ns	11 ± 5 days

LOS in hospitalized patients (∅ ± SD)	14 ± 11 days	.000	9 ± 5 days	.000	12 ± 9 days

Deaths within 30 days	54 (8.7%)	.000	2 (0.7%)	.000	234 (6.5%)

Patients with *Mycoplasma *pneumonia (MPP) were compared to patients with CAP due to other bacterial pathogens (p values marked as p^1^) and patients with CAP with unknown etiology (p values marked as p^2^), respectively. The significance level was set at < .001. (*) samples were available for standardized examination in the CAPNETZ central study unit. SD = standard deviation ns = not significant

## Results

### General characteristics of study population

Overall, 4532 patients with CAP from twelve clinical centers throughout Germany were included in our analysis. The 2492 male and 2040 female patients had a mean age of 60 ± 19 years. Sixty-five percent (n = 2922) of the patients were hospitalized when first contacted for participation in CAPNETZ. Co-morbidities were present in 2565 patients (57%). Thirty-one percent of the patients were smokers. Fifty-six percent of all patients presented with fever, 92% coughed, 73% had dyspnoea, and 8% showed signs of confusion. 106 patients (2.3%) required mechanical ventilation and 290 patients (6.4%) died (30 day mortality). The demographic and clinical data of the patients are given in Table 1.

### Diagnosis and epidemiology of *Mycoplasma pneumonia*

Respiratory samples were available from 1538 (33.6%) of the patients (Table 2). 148 (9.6%) of patients with respiratory samples had a positive *Mycoplasma pneumoniae *PCR.

**Table 2:** Diagnosis of Mycoplasma pneumonia in 4532 adult patients with community acquired pneumonia

Method	Samples tested	Samples with positive test result
PCR from respiratory material	1538	148 (9,6%)

Sputum	1335	134 * (10,1%)

Broncho-alveolar lavage	67	8 (11,9%)

Others (Throat washings)	136	6** (4,4%)

		

IgM-Antibody EIA	4450	204 (4,6%)

IgA-Antibody EIA	4450	881 (19,8%)

IgG-Antibody EIA	4450	1042 (23,4%)

		

PCR from respiratory material + serum	1448	
PCR positive + IgM-antibody positive		46 (3.2%)
PCR positive + IgA-antibody positive		55 (3.7%)
PCR positive + IgG-antibody positive		69 (4.7%)

PCR from respiratory material + serum	1448	
PCR negative + IgM-antibody positive		31 (2.1%)
PCR negative + IgA-antibody positive		246 (17%)
PCR negative + IgG-antibody positive		272 (18.8%)

* 5 samples were inhibited

** 2 samples were inhibited

Acute serum samples were available in 4450 patients. Altogether 204 (4.6%) patients had *Mycoplasma pneumoniae*-specific IgM antibodies in the acute phase serum sample. 46 patients were positive in both PCR and IgM antibody assay. Thus, 159 additional cases of *Mycoplasma pneumoniae *infection were identified by IgM antibody detection.

Taken together, 307 patients (6.8%) were considered as having definite *Mycoplasma *pneumonia (Table 2).

In support of our working definition, patients with a positive PCR from respiratory samples had almost identical characteristics if compared to patients with a positive IgM serum test, whilst patients with a positive IgA or IgG serum test were older, predominantly men, more often hospitalized, had higher CRB-65 scores and a fatality rate of 7.5% and 6.3%, respectively (Table 3).

**Table 3:** Comparison of Mycoplasma pneumonia with positive PCR from respiratory samples to patients with positive serology results

Variables	PCR positive n = 148	Only IgM positive n = 159	p-value^1^	Only IgA positive n = 826	p-value^2^	Only IgG positive n = 973	p-value^3^
Age ≥ 50 years	23.6%	23.3%	.841	67.6%	**.000**	60.5%	**.000**

Male gender	45%	38%	.240	58%	.003	54%	.026

Smoker	29%	42%	.017	37%	.037	34%	.243

Initial treatment setting							

-outpatient	66%	46%	**.000**	33%	**.000**	39%	**.000**

-hospitalized	34%	54%		67%		61%	

Fever	68%	59%	.081	55%	.003	56%	.004

Confusion	3%	2%	.632	9%	.013	8%	.023

CRB 65 Score							

0	74%	70%	.499	41%	**.000**	46%	**.000**

1–2	22%/4%	29%/1%		42%/13%		38%/12%	

3–4	0	1%/0		2%/1%		3%/1%	

Mechanical ventilation	0	1 patient	.332	23 patients	.040	20 patients	.078

Died within 30 days	1 patient (0.7%)	1 patient (0.6%)	.963	62 patients (7.5%)	.002	61 patients (6.3%)	.006

Patients with positive PCR were compared to IgM only (column p-values^1^) positive patients, to patients with only positive IgA (column p-values^2^) and patients with positive IgG only (column p-values^3^), respectively. Forty-six patients were positive in PCR and IgM, 55 patients were positive in PCR and IgA and 69 patients were positive in PCR as well as in IgG.

### Clinical characteristics of patients with CAP caused by *Mycoplasma pneumoniae*

**Figure 1:**
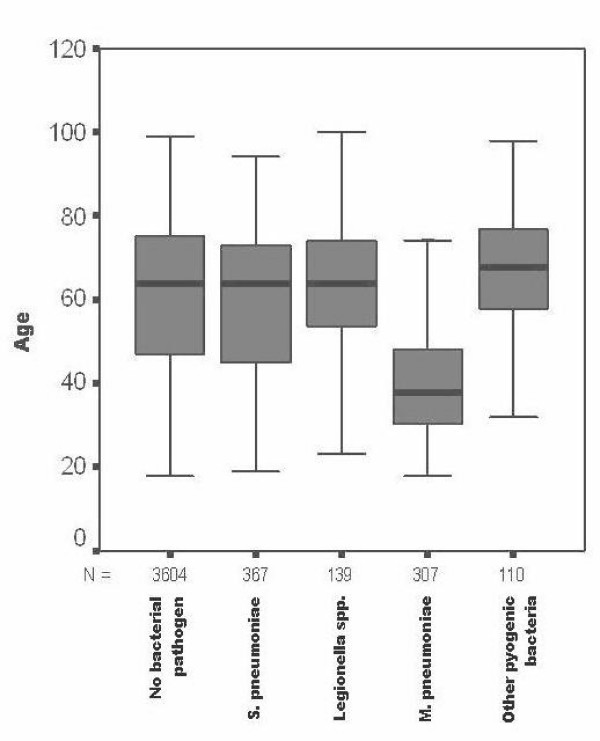
**Age distribution in 4527 patients with CAP**. The data for five patients with CAP due to *Chlamydophila pneumoniae *and a mean age of 36.8 ± 6.8 years are not shown in this graph.

Patients with *Mycoplasma *pneumonia were significantly younger than any other group of CAP patients with definite or unknown etiology in the study population (Figure 1) and clearly had fewer co-morbidities. The severity of their pneumonia was much lower as reflected by a high number of patients with low CRB-65 scores and a significantly lower inflammatory response as reflected by median leukocyte counts and CRP values on admission (Table 1). There were no statistically significant differences for the variables smoker, fever, cough, new auscultatory findings or chronic liver disease. In addition, though not statistically significant only one patient had to be mechanically ventilated and the length of hospitalization was significantly lower. The fatality rate was very low (0.7%) and significantly different if compared to the groups with another definite or unknown etiology, respectively.

Mixed infections of *Mycoplasma pneumoniae *with other bacterial pathogens were present in 20 cases; in particular *Legionella spp*. (n = 8), *Streptococcus pneumoniae *(n = 7), *Haemophilus influenzae *(n = 3), and *Staphylococcus aureus *(MSSA) (n = 2). None of the patients with mixed infection died.

Of 307 patients with *Mycoplasma *pneumonia, 171 were treated as outpatients and 136 were hospitalized. The former group was even younger, had very mild pneumonia, had fewer diabetic patients and a milder inflammatory response. No outpatient died (Table 4).

**Table 4:** Comparison of outpatients and hospitalized patients with *Mycoplasma pneumoniae *pneumonia

Variable	Outpatients N = 171	p value	Hospitalized patients N = 136
Age [∅ ± SD]	38 ± 13.1	**.000**	45 ± 18.4

Male gender	44%	.320	38%

CRB – 65 score			

0	79%	**.002**	64%

1 – 2	20%/1%		30%/5%

3 – 4	0		1% - 0

Smoker	39%	.185	32%

COPD	18%	.239	23%

Diabetes	0.6%	.013	5.1%

Malignancy	2%	.946	2%

			

CRP median (mg/l)	33	**.000**	90

Leucocytes median (10^3^/μl)	7900	**.000**	10300

			

Respiratory sample available	68%	**.000**	46%

Serum available	98%	.112	94%

			

Received antibiotic therapy active against atypical bacteria	64%	.306	70%

Length of antimicrobial therapy (days)	10.6 ± 4.1	.026	11.6 ± 3.6

Dyspnoea	60%	**.000**	80%

O 2 application	2%	**.000**	55%

Died (30 day mortality)	0	.112	2 patients

### Antimicrobial treatment and outcome of patients with *Mycoplasma pneumonia*

Detailed data concerning antimicrobial treatment were available for 97% of all patients. The predominant classes of antimicrobial agents administered were macrolides/ketolides (37%) and fluoroquinolones (29%). Overall, 65% of patients with *Mycoplasma *pneumonia had received antimicrobial agents active against atypical bacteria. There was a trend for patients with *Mycoplasma *pneumonia receiving more often antimicrobial agents covering atypical bacteria than those with another bacterial or without established etiology. There was no statistically significant difference in outcome (p = .11) or length of hospitalization (p = .057) between patients with *Mycoplasma *pneumonia who received appropriate or inappropriate antibiotic therapy, respectively.

A 71-years old patient diagnosed by PCR died several hours after he had been admitted to the hospital. The cause of death was not established. An additional female patient, aged 69 years, died after 10 days of hospitalization. She suffered from COPD, was a heavy smoker and had to be mechanically ventilated. Both patients had not received antibiotics active against atypical bacteria.

## Discussion

The main findings of our study, based on very strict diagnostic criteria in a large population of patients with CAP, can be summarized as follows: 1) *Mycoplasma pneumoniae *is an important pathogen causing CAP 2) patients with MPP are characterized by a quite specific clinical pattern, including younger age, absent or limited co-morbidity, limited inflammatory response, and usually presented with a mild to moderate pneumonia; 3) as a consequence, the majority of patients were treated as outpatients, hospitalized patients had a shorter length of stay, and mortality was minimal; 4) patients with *Mycoplasma *pneumonia treated as outpatients had even milder pneumonia than those hospitalized; 5) although there was a trend for patients with *Mycoplasma *pneumonia receiving antimicrobial drugs active against atypical bacterial pathogens more frequently than those with other or unknown etiologies, the rate of discordant treatment remained high.

This study is unique for the strict criteria for the diagnosis of *Mycoplasma *pneumonia and the large number of patients identified by this diagnostic approach. It is also unique for offering an identical extensive microbiological workup for hospitalized as well as outpatients. The incidence of *Mycoplasma pneumoniae *pneumonia was 6.7%, which is at the lower range of previous figures reported in recent large etiologic studies.

Previous series of patients with CAP due to *Mycoplasma pneumoniae *have largely relied on serologic testing, either using paired serum samples or including IgM and/or acute IgG or IgA [9-14]. When designing our study it was decided – due to feasibility – to collect only one acute phase serum sample. This might be considered as a limitation of our study.

When considering our study patients with *M.pneumoniae *pneumonia we felt safe to designate patients with a positive PCR result from respiratory samples as a proven case of *M.pneumoniae *infection. Due to the fact that in many cases acute phase sera were available and serological tests had been performed, we then had a closer look at our serological test results to answer the question whether patients with a positive IgM for *M.pneumoniae *in their acute phase serum might be considered as proven cases of *M.pneumoniae *infection as well. In fact, the detection of specific IgM antibodies is generally accepted as an indication of a recent infection. Two aspects persuaded us to follow this hypothesis:

(i) IgM antibodies to *M.pneumoniae *are only very rarely detected in the sera of healthy subjects. When evaluating the Virotech kit used in our study in blood donors and orthopaedic patients only 2 out of 602 patient samples were IgM positive (0.3%), whereas IgA and IgG antibodies were detected in a significant number of healthy persons [[15], C. Lück, personal communication].

(ii) Patients with a positive PCR result had very similar demographic and clinical characteristics if compared to patients with IgM antibodies only. Especially if looking at the initial CRB65 score, the proportion of patients requiring mechanical ventilation and the outcome (Table 3), we assumed it might be justified to regard PCR as well as IgM only positive patients as an entity if discussing clinical characteristics and management.

We used a commercially available test that uses specific *Mycoplasma pneumoniae *proteins as antigens. Therefore, a high specificity might be assumed and was demonstrated in a recent publication [15]. The detection of *Mycoplasma pneumoniae *DNA has a high positive predictive value. Albeit a persistence of *Mycoplasma pneumoniae *DNA after infection or within the incubation time has been reported it is generally accepted that such events are very rare (<0,5%) [16,17].

In one third of our MPP patients we found concordant positive PCR and positive IgM test results. The IgM EIA used showed a moderate sensitivity in sera collected in the acute phase [15]. Thus, it seems reasonable to assume that some of the PCR positive patients might not yet have developed IgM antibodies. On the other hand, PCR might have resulted false-negative due to the detection limit of the PCR detection as well as the rapid elimination of *Mycoplasma pneumoniae *after the initiation of antimicrobial treatment. In this context it should be noted that 27% of our patients received antibiotic agents at the time of inclusion into our study. The affection of antimicrobial treatment on clinical presentation and PCR or IgM responses in CAP caused by *M.pneumoniae *has never been studied to our knowledge. Therefore, we ignore the true effect of this confounder.

In contrast, clinical characteristics of patients with positive IgA titers were similar to that of patients with other bacterial and unknown etiologies but clearly different from the population with positive PCR and/or IgM, indicating that positive IgA titers represent persisting titers after infection occurring at any time. Moreover, the high prevalence of IgA antibodies (5–8%) found in blood donors and patients without respiratory symptoms strongly indicates a poor specificity. Therefore, IgA antibody detection is of very limited use as a diagnostic tool of pneumonia due to *Mycoplasma pneumoniae*.

Despite our different and strict diagnostic approach based exclusively on real-time PCR in respiratory samples and acute phase IgM, our data confirm previous findings of MPP being associated with several peculiar clinical characteristics. Apart from *Chlamydophila pneumoniae, Mycoplasma pneumoniae *is the only bacterial respiratory pathogen clearly occurring more frequently in younger adults. In a large study on the etiology of CAP, *Mycoplasma pneumoniae *was the only age-associated pathogen [18]. It appears that it usually occurs before the forth decade. In this age class, it has been shown that *Mycoplasma pneumoniae *can result as the most frequent pathogen even prior to *Streptococcus pneumoniae *[9,19]. Probably as a consequence, patients with *Mycoplasma pneumoniae *have considerably fewer co-morbidities. The only concomitant disease occurring with a frequency of more than 10% in our MPP population was COPD.

Another important feature of MPP is its usually less severe presentation, both in terms of clinical CRB-65 severity scores as well as inflammatory response. None of the severe complications of *Mycoplasma pneumoniae *reported in the literature could be observed in our series [2], although it cannot be definitely excluded that we missed single cases with severe *Mycoplasma *pneumonia. In accordance with the regularly mild presentation of MPP, and in line with several previous reports, more than half of our patients were treated as outpatients, hospitalized patients had a shorter length of hospitalization, and mortality was very low [10-14]. Moreover, those treated as outpatients were even younger and presented with a milder pneumonia than those hospitalized (with 99% having CRB-65 scores of less than 2).

Despite a trend for patients with MPP to receive more frequently antimicrobial treatment covering atypical bacterial pathogens, indicating that clinicians may have been aware of a probable *Mycoplasma *pneumonia, the rate of discordant treatment was high. Discordant treatment had no discernable effect on outcomes such as length of stay and mortality, indicating that MPP is usually a mild and self-limiting disease. Of note, however, both patients who died with MPP had received discordant treatment initially.

Our findings may have significant implications for future recommendations of empiric antimicrobial treatment in patients with CAP, particularly with respect to the need for covering atypical pathogens. If we consider *Legionella spp., Mycoplasma pneumoniae and Chlamydophila pneumoniae *as the three atypical bacterial pathogens treatable by antibacterial agents, it appears that only *Legionella spp*. are associated with a relevant mortality. In a recent series from our group, we could show that *Legionella spp*. was found with equal frequency in both ambulatory and hospitalized patients. However, severity was low and mortality was zero in ambulatory patients [5]. As a result, and in line with a recent meta-analysis of outcomes in non-severe CAP, atypical coverage does not seem to be relevant in terms of prevention of mortality in outpatients with CAP [20]. Following the definition of CAP used in our study we would advocate that in Germany as well as in other countries with comparable epidemiological settings a dual treatment with coverage of atypical pathogens as empirical standard therapy is not indicated for patients with mild CAP. However, antibiotic pneumococcal coverage continues to be the main demand in the treatment of this patient group.

Only recently, in a large study across four important world regions, Arnold et al. found a lower mortality in hospitalized patients receiving atypical coverage. As a result, and referring to several other studies with similar findings, they strongly recommended such coverage in all hospitalized patients [3,21,22]. In their study, the global incidence of *Legionella spp*. was 5%, of *Chlamydophila pneumoniae *7%, and of *Mycoplasma pneumoniae *12%. However, the rate of patients discharged alive at 14 and 30 days was not different but appeared only significantly different when the total number of patients discharged alive was considered, hinting at non-pneumonia-related reasons for different outcomes. Several other reports also do not support the conclusion of Arnold et al. [20,23-25]. Taking into account these reports and the present data, increased mortality in hospitalized patients with CAP is almost exclusively related to cases with moderate to severe pneumonia caused by *Legionella spp*. Another concern relates to mixed infections. In our series, the rate of mixed infections was low (n = 20), with no associated mortality. Most co-infections were caused by pneumococci and *Legionella spp*. which may be detected using routine diagnostic methods according to current ATS/IDSA recommendations [26]. Thus, a strategy of active search for patients with *Legionella spp*. or, in cases of more severe pneumonia, also for patients with *Mycoplasma pneumoniae *and *Chlamydophila pneumoniae *should obviate the need of regular atypical coverage in all hospitalized patients.

At present, we suggest that in hospitalized patients with CAP coverage of atypical bacteria can be limited to patients with severe CAP, those with confirmed legionellosis, *Chlamydophila pneumoniae *pneumonia and those with probable MPP, i.e. younger patients (age < 40 years) with absent or mild co-morbidity and a mild clinical presentation (CRB-65 < 2).

## Conclusion

In conclusion, our study confirms previous reports about characteristic patterns of CAP through *Mycoplasma pneumoniae*. However, realizing the minimal risk of adverse outcomes associated with this condition, and having in mind recent findings of our group on legionellosis, atypical coverage of all patients presenting with mild CAP as defined in our study seems questionable. Instead, our data provide important hints for strategies aimed at a more judicious use of broad spectrum antimicrobial treatment with atypical coverage.

## Competing interests

The authors declare that they have no competing interests.

## Authors' contributions

HvB carried out analysis and interpretation of the data, performed the statistical analysis, participated in the coordination of the study and drafted the manuscript. TW participated in the design, coordination and supervision of the study as well as analysis and interpretation of the data, RM participated in the design, coordination and supervision of the study as well as analysis and interpretation of the data. NS participated in the design, coordination and supervision of the study. CL carried out all molecular and serological studies and participated in the analysis and interpretation of the data. SE participated in the analysis and interpretation of the data and drafted the manuscript. All authors read and approved the final manuscript.

## Pre-publication history

The pre-publication history for this paper can be accessed here:

http://www.biomedcentral.com/1471-2334/9/62/prepub
